# Improved quality of life with home therapy with subcutaneous immunoglobulins for patients with secondary hypogammaglobulinaemia

**DOI:** 10.1186/1710-1492-8-S1-A26

**Published:** 2012-11-02

**Authors:** Marie-Claude Levasseur, Élie Haddad

**Affiliations:** 1 CHU Sainte-Justine, 3175 Cote Sainte-Catherine, Montreal, QC, Canada H3T 1C5

## Background

We reported 10 cases where SCIg turned out to be the preferred route of Ig replacement therapy for patients who developed secondary hypogammaglobulinaemia following bone marrow transplantation or chemotherapy treatment for neoplasia. Patients’ age ranged from 2 to 19 years old, 7 patients being below the age of 10. Six out of the 10 patients were transitioned from IVIg to SCIg: 4 patients due to systemic side effects associated to IVIg perfusions and 2 patients due to poor quality of life related to frequent travels to the hospital for IVIg infusion. Four out of the 10 patients were Ig naïve patients and were started directly on SCIg. All patients received a weekly dose of 100mg/Kg infused in two different sites assisted by a battery-powered pump. Patients were given a 1:1 dose when transitioned from IVIg to SCIg. A significant increase of the IgG level was noted in all patients. All patients tolerated very well SCIg therapy with no reported systemic adverse reactions. Local infusion sites reactions were the most frequent observed reactions with a frequency similar to the one observed in patients with primary immunodeficiency disorders (PIDD). Lastly, parents reported improvement in quality of life under SCIg therapy; their child had improved social functioning, better resistance against infections and much improved overall health.

## Conclusion

While home-based SCIg administration relative to hospital-based IVIg has been shown to improve PIDD patients’ quality of life, we believe that SCIg has the potential to improve patient’s satisfaction and independence also in patients with secondary hypogammaglobulinemia.

